# Supplementary material for: Modeling and comparing data mining algorithms for prediction of recurrence of breast cancer
Source: PLoS One. 2020 Oct 15;15(10):e0237658. doi: 10.1371/journal.pone.0237658 (PMC7561198; doi:10.1371/journal.pone.0237658)
Supplement: Attachment — Submitted filename: Response to Reviewers-v12.docx [file pone.0237658.s002.docx]

**Response to the Reviewers**

**Dear Editor-in-Chief,**

We wish to thank you, the associate editor, and the reviewers for the comments we received on the attached paper, and also thank you for giving us the opportunity to revise the manuscript again Which have helped to enhance the quality of the paper. We hereby submit a revised version of the paper with ID number of PONE-D-19-19077. Significant changes and modifications were highlighted in the revised manuscript, and the detailed responses to reviewers’ comments are listed as follows. We hope that the modified version is acceptable, and we look forward to your kind recommendations.

Best regards,

The Authors

**Editor Comments to the Author**

**Comment 1:**

One reviewer points out that clinical applicability of the results remains vague. Although PLOS ONE's publication criteria do not include perceived significance, if the manuscript does not aim to directly address clinical applications, this should be more clearly stated.

**Response and corrections regarding comment 1:**

Thank you for your positive view. According to your valuable comment, all the manuscript is fully revised and the main aims of this research, that is prediction of breast cancer recurrence in large scale dataset with comparing data mining algorithms and presenting clinical suggestions, precisely is stated and explained more clearly in introduction and conclusion and discussion (please see section “**Introduction**” on pages 1-3 and section “**Conclusion and Discussion**” on page 18).

**Comment 2:**

In ethics statement in the manuscript and in the online submission form, please provide additional information about the database used in your retrospective study. Specifically, please ensure that you have discussed whether all data were fully anonymized before you accessed them and/or whether the IRB or ethics committee waived the requirement for informed consent. If patients provided informed written consent to have their data used in research, please include this information.

**Response and corrections regarding comment 2:**

Accordance with your helpful comment, ethics statement added at the end of the manuscript that explained additional information about the database used in our research (please see section “**Ethics Statement**” on page 19).

**Comment 3:**

The manuscript must describe a technically sound piece of scientific research with data that supports the conclusions. Experiments must have been conducted rigorously, with appropriate controls, replication, and sample sizes. The conclusions must be drawn appropriately based on the data presented:

**Response and corrections regarding comment 3:**

We highly appreciate this comment. According to your helpful comment, the scientific process of conducting the research and writing the manuscript is fully revised. In the introduction we have added the aim of this study, the importance of it and the problem that we're looking for solving it (please see section “**Introduction**” on pages 1-3). After library studies, field surveys, and interviews with physicians and breast cancer specialists, 23 primary factors affecting **breast cancer recurrence** are identified, and in the next step, with chi-square test and the p-value measured based on the dataset, eight key factors affecting the recurrence of breast cancer are extracted from the Iranian Ministry of Health and Medical Education as well as the Cancer Research Center(please see section “**Materials and Methods**” on page 3 and Table 5 on page 8). Then, seven conventional and new machine learning algorithms are used to predict the recurrence of breast cancer. In this step, analyzing is conducted rigorously, with appropriate controls, replication, and sample sizes that implicated in explanation of each algorithms., (please see the last paragraph of page 4 and explanations of pages 9-16)and at the final step of research, Based on the results of analyzing a dataset, practical suggestions and conclusions are drawn, added and presented (please see section “**Conclusion and Discussion**” on page 18).

**Comment 4:**

PLOS ONE does not copyedit accepted manuscripts, so the language in submitted articles must be clear, correct, and unambiguous. Any typographical or grammatical errors should be corrected at revision, so please note any specific errors here.

**Response and corrections regarding comment 4:**

Based on this useful comment, we carefully have read, revised, and rewritten the whole text of the paper again and made essential corrections, and the manuscript is edited for proper English language, grammar, punctuation, spelling, and overall style.

**Review Comments to the Author:**

**Reviewer 1 Comments:**

The use of data mining in the clinical setting is a powerful tool and should be explored.

However,

**Comment 1:**

the clinical scenario proposed, breast cancer, is far too complex than "benign breast cancer" and "malignant breast cancer." Moreover, these entities are not clearly defined. There are no info about TNM, Her2, Estrogen receptors, subtitles (TNBC, HER2 status, ER+/-, etc.), follow up, treatment, etc.

**Response and corrections regarding comment 1:**

Based on your precise and valuable comment, in the revised manuscript, the information about TNM, Her2, Estrogen receptors, subtitles (TNBC, HER2 status, ER+/-, etc.), follow up, treatment, etc. is added to our results (Please see Section “**Results**” on pages 5-9 and Tables 2, 3, 4 and 5 in this section).

**Comment 2:**

although the methodology could be useful, this work has no impact regarding the clinical scenario, is merely the application of three algorithms to predict a binary classification using ten variables with a huge amount of cases.

A more complex clinical scenario must be evaluated to ensure that these algorithms can help to solve true clinical needs.

**Response and corrections regarding comment 2:**

Based on this helpful and valuable comment, we have changed the clinical scenario of our research from a form of diagnosis of benign or malignant based on ten manifestations of the breast to the modeling and comparing data mining algorithms for prediction of **recurrence of breast cancer**.

Precise disease forecasting tools and mechanisms along with physicians' experience, can play a practical role in the correct diagnosis and treatment choice be it and also a matter of confidence in the accurate diagnosis of the disease both the physician and the patient favorably increased. On the other hand, how the diagnosis will determine the next steps for the patient. So, if it is of low accuracy, it affects one's survival, and it is vital to have excellent and acceptable accuracy of forecasting models.

Artificial neural networks can be used as a diagnostic method with high sensitivity and specificity to detect prediction of recurrence of breast cancer tumors, besides other non-invasive diagnostic methods (such as mammography and radiography). The importance of this result is that it can prevent patients, who do not need any invasive diagnostic methods (sampling and surgery), from such operations. In addition to the decrease in the costs, Faster and more precise diagnostic of prediction of recurrence of breast cancer may increase the treatment chance (please see page 2, paragraph numbers of 4-6., and page 3 paragraph number of 1 and 2).

This study is performed among patients with breast cancer who had been followed for at least two years from June 2018 to June 2019. The data collection is conducted using the official statistics of the ministry of health and medical education, and Iran Cancer Research Center. Required data are collected from patients' records, including the status of 23 Variable.

After data gathering and Extraction of key factors that are critical for the prediction of recurrence of breast cancer, we compared 7 Data Mining Algorithms and have discuss and conclude practical suggestions to help to the prediction of recurrence of breast cancer that is one of the important clinical needs(please see section “**Materials and Methods**” on page 3 and first and second paragraph of section “**Results**” on page 5 and 6).

**Reviewer 2 Comments:**

The authors compared 3 data mining algorithms' performance on a large-scale breast cancer dataset. The data of this study was obtained officially from the Ministry of Health and Medical Education and the Iran Cancer Research Center. Its database included 10337 independent records, which can be considered as one of the largest breast cancer datasets. The research can help doctors without sufficient diagnostic facilities to make more accurate diagnose in remote areas.
However, there are still some shortcomings and limitation in this work:

**Comment 1:**

All the three machine learning algorithms are relatively out-of-date. All these three algorithms are classic machine learning algorithms. However, authors should conduct the comparison against more advanced and competitive methods, since the scale of dataset is quite large, a lot of recent methods can be fairly compared without the risk of overfitting. Contribution is limited. From my point of view, the dataset in this work has quite a good scale and quality. However, the following experiments did not make full use of the dataset. No new breast cancer prediction method or valuable selection strategies over different machine learning algorithms were proposed."

**Response and corrections regarding comment 1:**

Thanks for your positive view of our paper. According to your valuable comment, we have tried to make better use of the dataset and better research contribution. So, the present research scenario is changed from predicting benign or malignant to predicting recurrence of breast cancer based on eight key factors. After library studies, field surveys, and interviews with physicians and breast cancer specialists, 23 primary factors affecting **breast cancer recurrence** are identified, and in the next step, with chi-square test and the p-value measured based on the dataset, eight key factors affecting the recurrence of breast cancer are extracted from the Iranian Ministry of Health and Medical Education as well as the Cancer Research Center official dataset(please see section “**Materials and Methods**” in page 3 and Table 5 on page 8). Then, according to your helpful comment, we add four conventional and newer algorithms C5.0, Random forest, and KPCA-SVM, SVM to our analyzing and with 7 data mining algorithms we analyze our dataset. (please see Paragraph 5 on page 3, the section of “**Data mining methods”** on page 4**.,** Paragraph 2 on page 12, Section of **“Modeling using KPCA- SVM”** on page 12**.,** and also explanations of Pages 13-17**)**

**Comment 2:**

The author simply used accuracy to measure different kind of methods. It is not sufficient. Obviously, the misdiagnosis of cancer will cause more serious consequences in actual situations. It is recommended to use the F-score or area under the ROC (Receivers Operating Characteristic) curve as the measure to evaluate and compare performances of different algorithms.

**Response and corrections regarding comment 2:**

Based on this helpful comment the results are compared based on TP, T indices N, FP, FN, Accuracy, Sensitivity, Specificity, The Geometric mean of sensitivity and specificity, PPV, NPV The Geometric mean of PPV and NPV, F-measure and the area under ROC curve (please see page 16 section of “**Performance metrics,"** and also table 9, figure 3 and the last paragraph on page 17).

Finally, the authors are thankful to the editors and reviewers for the critical comments and constructive suggestions, which helped improve the quality and presentation of the paper significantly.
